# Is duodeno-jejunal bypass liner superior to pylorus preserving bariatric surgery in terms of complications and efficacy?

**DOI:** 10.1007/s00423-021-02131-x

**Published:** 2021-03-12

**Authors:** Istvan Bence Balint, Ferenc Csaszar, Krisztian Somodi, Laszlo Ternyik, Adrienn Biro, Zsolt Kaposztas

**Affiliations:** 1 Department of Surgery and Vascular Surgery, Zala County Saint Rafael Hospital, H-8900 Zrinyi Miklos street 1., Zalaegerszeg, Hungary; 2 Doctoral School of Neurosciences, University of Pecs, Pécs, Hungary; 3 Department of Surgery, Somogy County Kaposi Mor Teaching Hospital, Kaposvár, Hungary

**Keywords:** Pylorus preserving, Bariatric surgery, Metabolic surgery, Single-anastomosis duodeno-jejunal bypass, Single-anastomosis duodeno-ileal bypass, Duodenal switch, Duodeno-jejunal bypass liner, EndoBarrier

## Abstract

**Purpose:**

Based on recent scientific evidence, bariatric surgery is more effective in the management of morbid obesity and related comorbidities than conservative therapy. Pylorus preserving surgical procedures (PPBS) such as laparoscopic single-anastomosis duodeno-jejunal or duodeno-ileal bypass with sleeve gastrectomy are modified duodenal switch (DS) surgical techniques. The duodeno-jejunal bypass liner (DJBL) is a novel surgical method in the inventory of metabolism focused manual interventions that excludes duodeno-jejunal mucosa from digestion, mimicking DS procedures without the risk of surgical intervention. The aim of this article is to summarize and compare differences between safety-related features and weight loss outcomes of DJBL and PPBS.

**Methods:**

A literature search was conducted in the PubMed database. Records of DJBL-related adverse events (AEs), occurrence of PPBS-related complications and reintervention rates were collected. Mean weight, mean body mass index (BMI), percent of excess of weight loss (EWL%), percent of total weight loss (TWL%) and BMI value alterations were recorded for weight loss outcomes.

**Results:**

A total of 11 publications on DJBL and 6 publications on PPBS were included, involving 800 and 1462 patients, respectively. The baseline characteristics of the patients were matched. Comparison of DJBL-related AEs and PPBS-related severe complications showed an almost equal risk (risk difference (RD): −0.03 and confidence interval (CI): −0.27 to 0.21), despite higher rates among patients having received endoscopic treatment. Overall AE and complication rates classified by Clavien-Dindo showed that PPBS was superior to DJBL due to an excess risk level of 25% (RD: 0.25, CI: 0.01–0.49). Reintervention rates were more favourable in the PPBS group, without significant differences in risk (RD: −0.03, CI: −0.27 to 0.20). However, PPBS seemed more efficient regarding weight loss outcomes at 1-year follow-up according to raw data, while meta-analysis did not reveal any significant difference (odds ratio (OR): 1.08, CI: 0.74–1.59 for BMI changes).

**Conclusion:**

Only limited conclusions can be made based on our findings. PPBS was superior to DJBL with regard to safety outcomes (GRADE IIB), which failed to support the authors’ hypothesis. Surgical procedures showed lower complication rates than the incidence of DJBL-related AEs, although it should be emphasized that the low number of PPBS-related mild to moderate complications reported could be the result of incomplete data recording from the analysed publications. Weight loss outcomes favoured bariatric surgery (GRADE IIB). As the DJBL is implanted into the upper gastrointestinal tract for 6 to 12 months, it seems a promising additional method in the inventory of metabolic interventions.

## Introduction

### Rationale

Obesity represents a high risk for metabolic syndrome-related morbidities, such as hypertension, dyslipidaemia, prediabetes (hyperinsulinemia, impaired fasting glucose) and type II diabetes mellitus, resulting in various forms of cardiovascular disease [1, 2]. According to recent scientific evidence, bariatric surgery is the most efficient method to obtain weight loss. However, there is significant difference regarding complications and weight loss outcomes, depending on the type of surgical method [3–6].

Pylorus preserving surgical procedures (PPBS) date back to the early 1990s and have advantages over gastric bypass procedures (laparoscopic Roux-en-Y gastric bypass and one-anastomosis gastric bypass, LRYGB and OAGB, respectively) due to the preservation of the pylorus by a tube-like stomach (gastric sleeve), resulting in controlled gastric emptying and prevention of afferent limb bile reflux. The single-anastomosis duodeno-jejunal and duodeno-ileal bypass with sleeve gastrectomy (SADI-SG and SADJ-SG, respectively) are the most frequently applied methods of PPBS. These procedures are variants of the duodenal switch (DS) technique, representing favourable efficacy with acceptable complication rates, and vary in applicable technique. When using SADI-SG, a part of the ileum (200–300 cm measured backwards from the ileocecal valve) is connected to the duodenal stump after performing laparoscopic sleeve gastrectomy (LSG). Identical to OAGB, the jejunum (150–200 cm measured downwards from the ligament of Treitz) is used to create the duodeno-jejunal anastomosis in SADJ-SG. Both methods have a similar effect on weight loss and metabolic improvements, with affordable complication rates [7–12].

The duodeno-jejunal bypass liner (DJBL) (EndoBarrier®, GI Dynamics, Boston, MA, USA), introduced in the late 2000s, is a novel investigational method among metabolic interventions. After initial FDA approval, it was still not widely used for years because of severe complications, such as liver abscess and pancreatitis. A 60-cm-long impermeable fluoropolymer tube is inserted endoscopically under general anaesthesia into the duodenum and becomes anchored to the pylorus (the implant secures itself) in outpatient settings. It excludes the duodeno-jejunal mucosa from digestion mimicking DS procedures without the potential risk of surgery. Favourable weight loss outcomes and metabolic control are expected by creating a physical barrier between the mucosa of the upper small intestine and the ingested food. Longitudinal temporal data on efficacy is lacking, and published complication rates are controversial [13–23].

### Objective

The aim of this review article is to summarize and compare differences between the procedure-related complication rates and weight loss outcomes of DJBL and PPBS by performing a meta-analysis.

## Methods

### Study design

This systematic review including meta-analysis was registered under #CRD42020165718 in the PROSPERO registry and was conducted according to the PRISMA Statement. The study protocol is available at the website of National Institute for Health Research (https://www.crd.york.ac.uk/PROSPERO/).

#### Eligibility criteria

Studies (randomized controlled trials (RCTs), matched cohorts, case series) investigating DJBL and/or PPBS (SADJ-SG and/or SADI-SG) presenting adult patients (18–65-year age interval) with a body mass index (BMI) over 40, or over 35 if a metabolic indication was present, and at least 12-month follow-up after surgery and a 12-month planned and completed implantation period for DJBL were eligible. Papers presenting revisional procedures (presence of bariatric surgery in previous history) and those with sample sizes below 15 cases were excluded.

#### Information sources and literature search

PubMed was used, with keywords ‘endobarrier’, ‘duodenojejunal bypass liner’, ‘duodeno-jejunal bypass liner’, ‘duodeno jejunal bypass liner’, ‘gastrointestinal bypass liner’, ‘gastro intestinal bypass liner’, ‘gastro-intestinal bypass liner’, ‘single anastomosis duodeno ileal bypass’, ‘single anastomosis duodenoileal bypass’, ‘single anastomosis duodeno-ileal bypass’, ‘single-anastomosis duodenoileal bypass’, ‘single-anastomosis duodeno-ileal bypass’, ‘one anastomosis duodeno ileal bypass’, ‘one anastomosis duodenoileal bypass’, ‘one anastomosis duodeno-ileal bypass’, ‘one-anastomosis duodenoileal bypass’, ‘one-anastomosis duodeno-ileal bypass’, ‘single anastomosis duodeno jejunal bypass’, ‘single anastomosis duodenojejunal bypass’, ‘single anastomosis duodeno-jejunal bypass’, ‘single-anastomosis duodenojejunal bypass’, ‘single-anastomosis duodeno-jejunal bypass’, ‘one anastomosis duodeno jejunal bypass’, ‘one anastomosis duodenojejunal bypass’, ‘one anastomosis duodeno-jejunal bypass’, ‘one-anastomosis duodenojejunal bypass’ and ‘one-anastomosis duodeno-jejunal bypass’, without language restrictions and filters, to include studies on investigated methods until a publication date of 30th of March, 2020.

#### Study selection

After identifying publications through the database search, duplicates were removed. Through screening, some studies not meeting the eligibility criteria were excluded. The remaining articles were retrieved for complex evaluation. After removing full-text papers not meeting the eligibility criteria, studies were included into qualitative and quantitative analysis. Studies with overlapping records were excluded from the final evaluation.

#### Data collection

Outcomes of safety and weight loss were collected from individual studies after duplications were excluded.

#### Data items

The number of adverse events (AEs) of DJBL and complications (CD 1–5) of surgeries were collected for safety analysis. Mean weight, mean BMI, percent of excess of weight loss (EWL%), percent of total weight loss (TWL%) and changes of BMI at 1-year follow-up after initial intervention were recorded for weight loss outcomes. Categorical variables were presented as number and percentage. Continuous variables were presented by mean, range and SD, where possible.

#### Bias

A wide search without language restriction and filters was undertaken in an attempt to minimize selection bias. All available study types were included to increase the sample size, causing bias due to insecure parameters with weak statistical results. Heterogeneity tests (Cochran’s Q, *I*^2^ consistency and chi-square tests) were performed to verify validity (the *p* value was set at 0.05). Doi plots were presented to detect publication bias. The IVhet method was applied for meta-analysis to minimize underestimation of statistical error [24–28].

#### Summary measures

AEs and complications were compared by risk difference (RD) between the investigated methods. Odds ratios (ORs) are presented for weight loss outcomes (BMI comparison).

#### Synthesis of results

MetaXL software (ver. 5.3, additional software for Microsoft Excel, EpiGear International) was applied. The IVhet model was chosen for meta-analysis, which is an inverse variance method developed by Doi et al., to keep the coverage at the usual 95% level of confidence interval (CI) and maintain the inverse variance weights of the studies. In case of heterogeneity, the model boosts the CI around the pooled estimate, but the study weights remain individual depending on the size of the study population [24–28].

## Results

### Study selection

**Fig. 1:**
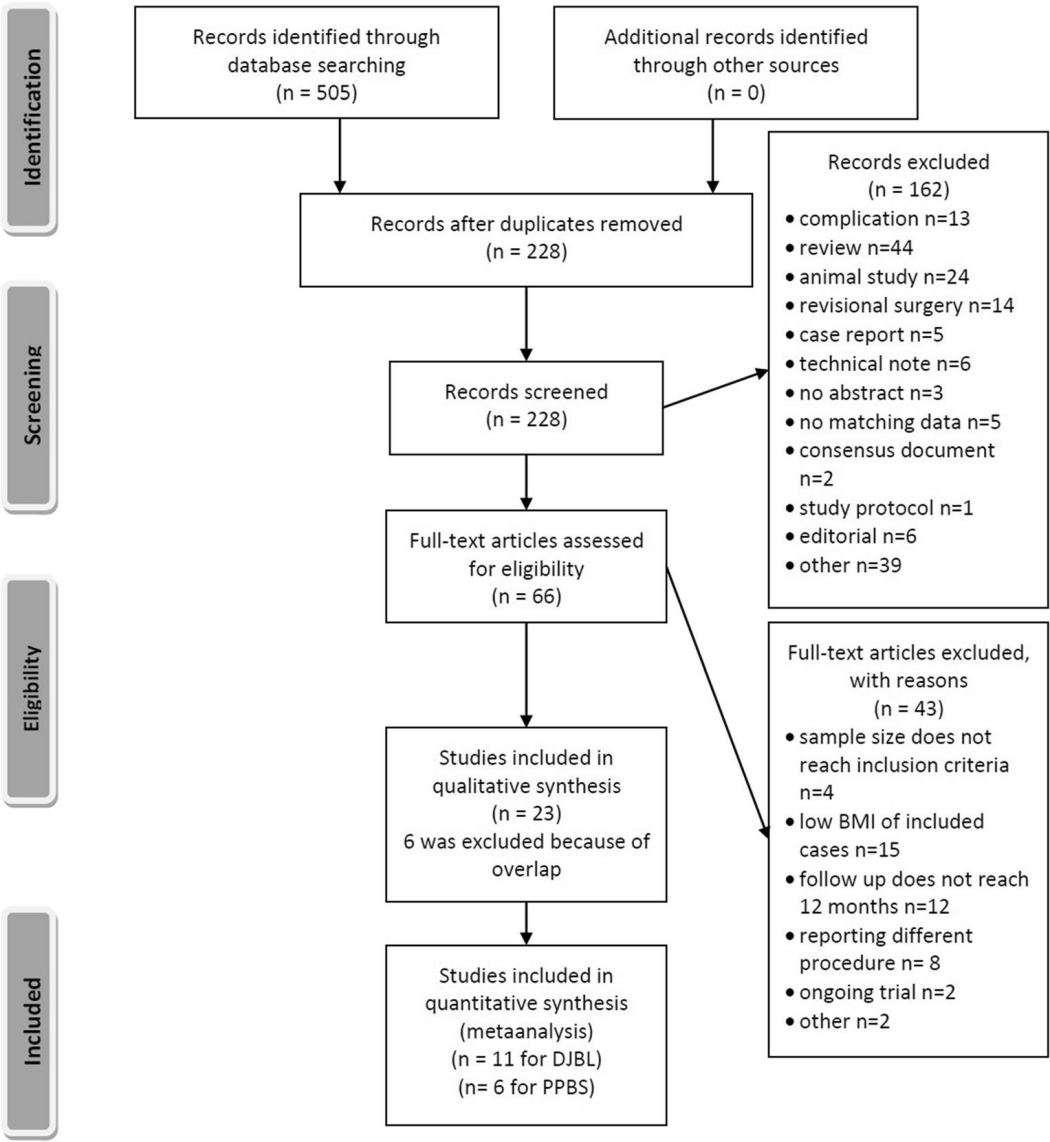
Flow diagram of studies

PubMed database analysis identified 505 unique publications, and 228 articles remained after duplications were removed. Overall, 23 studies met the eligibility criteria from the screened and assessed full-text publications. Due to overlap, 6 publications were excluded, leaving 17 publications in the final analysis (11 for DJBL and 6 for PPBS) [7–23]. Details are listed in the flow chart presented in Fig. 1.

### Study characteristics

In total, 7 single-centre prospective cohorts, 1 two-centre cohort and 3 multicentre cohorts were included for DJBL, resulting in 800 involved patients (Table 1), and 3 single-centre prospective cohorts, 1 two-centre cohort, 1 international retrospective mixed cohort and 1 small RCT were identified for PPBS, involving 1462 surgically treated cases (Table 1).

**Table 1:** Characteristics of studies

Authorship	Year of publication	Countrv	Study design	Method	Number of cases treated by the investigated method	Follow-up (implantation period + postimplantation period for DJBL)	Classification of complications by Clavien-Dindo (determined by authors)						
							CD1	CD2	CD3a	CD3b	CD4a	CD4b	CD5
Roehlen et al.	2020	Germany	Single-centre prospective cohort	DJBL	71	12 months	66	18	155	0	2	0	0
Homan et al.	2019	Slovenia	Single-centre prospective cohort	DJBL	19	12+12 months	14	0	33	1	1	0	0
Deutsch et al.	2018	Israel	Single-centre prospective cohort	DJBL	51	12+12 months	6	2	59	0	0	0	0
Laubner et al.	2018	Germany	Multicentre prospective matched cohort (DJBL and DPV registry)	DJBL vs. Conservative treatment (2:1)	235	12 months	81	11	327	0	0	0	0
Patel et al.	2018	United Kingdom	Multicentre prospective cohort	DJBL	45	12+6 months	98	33	176	1	0	0	0
Riedel et al.	2018	Germany	Multicentre prospective cohort (DJBL registry)	DJBL	66	12 months	28	5	99	0	0	0	0
Forner et al.	2017	Australia	Two-centre retro- and prospective cohort	DJBL	114	12+6 months	84	32	230	0	1	0	0
Quezada et al.	2017	Chile	Single-centre prospective cohort	DJBL	80	12-+24 months	37	8	125	0	2	0	0
Stratmann et al.	2016	Germany	Single-centre prospective cohort	DJBL	18	12 months	0	1	19	0	0	0	0
Munoz et al.	2013	Chile	Single-centre prospective cohort	DJBL	79	12 months	0	4	83	0	0	0	0
de Moura et al.	2012	Brazil	Single-centre prospective cohort	DJBL	22	12 months	33	5	60	0	0	0	0
					800		447	119	1366	2	6	0	0
Authorship	Year of publication	Country	Study design	Method	Number of cases treated by the investigated method	Overall follow-up	Classification of complications by Clavien-Dindo						
							CD1	CD2	CD3a	CD3b	CD4a	CD4b	CD5
Surve et al.	2020	Australia	Single-centre prospective cohort	SADI-SG	91	2 years	0	4	0	0	0	0	0
Surve et al.	2018	Multinational	International retrospective mixed cohort	SADI-SG	598	12 months	0	0	5	7	0	0	0
Zaveri et al.	2018	USA	Two-centre prospective cohort	SADI-SG	437	4 years	20	22	2	35	0	0	1 leak, 1 cardiac arrest, 1 sudden cardiac death probably due to OSA
Moon et al.	2017	USA	Single-centre prospective cohort	SADI-SG	140	2 years	0	28	0	17	0	0	1 (ventricular fibrillation)
Sanchez-Pernaute et al.	2015	Spain	Single-centre prospective cohort	SADI-SG	168	5 years	0	38	0	3	0	0	0
Raj et al.	2011	India	Single-centre RCT	SADJ-SG with DJ vs. RYGB	28	1 year	0	0	0	1	0	0	0
					1462		20	92	7	63	0	0	4

AE and complications were classified by Clavien-Dindo

### Risk of bias within studies

One retrospective study was involved for safety analysis because it presented controlled results of different intervention sites. Some patients of this international retrospective mixed cohort were excluded because their cases were described in more detail in other single-centre prospective cohorts involved in the analysis, and the other publications represented high quality-controlled data on safety and efficacy. All AEs were taken into consideration without subgroups. Complications of surgeries were essentially graded according to the Clavien-Dindo classification system [29, 30]. For better comparison, authors reconsidered AEs of DJBL using the Clavien-Dindo classification (CD 1, gastrointestinal tract (GIT) events; 2, cholangitis, anaemia, bleeding, malabsorption, diarrhoea, constipation and other difficulties; 3a, early removal, migration, hepatic abscess, perforations, erosion and obstruction; 3b, cholecystectomy; 4a, pancreatitis; 4b, no cases; 5, no cases). Three safety-related comparisons were performed (1, author defined severe events and complications; 2, overall number of AEs and CD complications; 3, reintervention rates). DJBL-related severe events included death, hepatobiliary complication, device migration, pancreatitis, mucosal injury, obstruction and bleeding. PPBS-related severe complications included venous thromboembolism (VTE), wound healing disorder, ileus, hepatobiliary complication, leakage, death, stenosis or stricture, conversion, intraabdominal abscess, bleeding, peritonitis, biliary reflux, weight regain, perforation, diagnostic laparoscopy and obstruction. Weight loss outcomes were presented by descriptive comparison of initial weight, BMI changes, EWL% and TWL% at 1 year after intervention. The meta-analysis was performed on BMI changes (mean differences with SD were estimated from individual studies and were compared to each other).

### Results of individual studies

In the 11 DJBL-related studies with 800 patients, the mortality rate was zero, a high rate of AEs (73.5%) was reported, and 19% of the implanted devices were explanted earlier than planned. The number of severe AEs was 155 (19.4%). In the 6 studies in the PPBS group, which included 1462 patients, 4 patients died: 1 death was a result of leakage and the other 3 deaths were not surgery related. Complications occurred at an acceptable rate (12.4% in all surgical cases), and 37 reoperations (2.5% of patients) were performed due to various reasons. In total, 5.7% (84) of all complications were listed in the severe category. AEs of DJBL and surgery-related complications are presented in Table 2 and Table 3, respectively.

**Table 2:** DJBL-related complications

Device-related AE rate	Death	Early removal	Migration	Hepatic abscess	Pancreatitis	Hepatobiliary	Anaemia	Bleeding	GIT events	Malabsorption	Diarrhoea	Constipation	Oesophageal perforation/rupture	Duodenal perforation	Mucosal erosion	Obstruction	Other technical difficulties	Other
78	0	20	4	3	2	1 cholangitis	0	0	66	0	11	6	0	1	0	1	0	0
16	0	0	0	0	1	1 cholangitis with cholecystectomy	0	0	14	0	0	0	0	0	0	0	0	0
20	0	10	0	2	0	0	0	2	6	0	0	0	0	0	0	0	0	0
107	0	35	6	4	0	1 cholecystitis	0	1	81	0	6	3	0	1	1	2	0	0
54	0	6	2	0	0	1 cholecystectomy	5	1	98	18	4	4	0	0	1	0	0	1
43	0	0	1	1	0	0	0	4	28	0	0	1	0	0	7	1	0	0
84	0	28	11	2	1	0	11	5	84	11	3	0	0	0	6	6	1	1
131	0	23	9	3	2	0	0	5	37	0	3	0	2	0	1	2	0	0
5	0	3	3	0	0	0	0	1	0	0	0	0	0	0	0	1	0	0
20	0	18	8	1	0	1 cholangitis 1 cholecystitis	0	1	0	0	0	0	0	0	0	5	0	1
30	0	9	3	0	0	0	0	1	33	0	1	0	0	0	0	0	0	3

**Table 3:** PPBS-related complications

Overall number of complications	Death	Reoperation	VTE	Leakage	Stricture	Common channel shortening	Common channel lengthening	Afferent limb reflux	Intraabdominal abscess	Bleeding	Wound infection	Constipation	Ileus	GIT events	Malabsorption	Diarrhoea	Small bowel perforation	Other technical difficulties	Other
4	0	0	0	0	0	0	0	0	0	2	0	0	0	0	0	0	0	0	1 euglycaemic diabetic ketoacidosis 1 respiratory insufficiency
12	0	0	0	6	5	0	0	1	0	0	0	0	0	0	0	0	0	0	0
79	1 leak, 1 cardiac arrest, 1 sudden cardiac death probably due to OSA	6	1 portal vein	1 early	13	0	8	4	2	6	11	1	0	23	2	9	0	3	0
45	1 (ventricular fibrillation)	27	1 DVT	5 duodeno-ileal anastomosis/3 sleeve/2 duodenal stump	0	0	0	2	1	0	0	0	2 internal hernia and twisting	0	28	0	1	0	2 diagnostic laparoscopy 1 gastroenteritis
41	0	3	0	1	0	0	0	0	0	1	0	0	0	0	38	0	0	0	1 umb. hernia
1	0	1	0	0	0	0	0	0	0	0	0	0	1	0	0	0	0	0	0

Mean weight and BMI at baseline were comparable between groups, while EWL%, TWL% and BMI at 1 year were in favour of the PPBS group (76.5% vs. 33.5% for EWL%, 36.9% vs. 13.7% for TWL% and a BMI decrease of 18 vs. 4.2, respectively). Records are presented in Table 4, and a summary of complications and weight loss outcomes is detailed in Table 5.

**Table 4:** Weight loss outcomes

Weight loss outcomes of DJBL				
Mean weight in kg at baseline	EWL% at 1 year	TWL% at 1 year	BMI in kg/m2 at baseline	BMI in kg/m2 at 1 year
n.a.	n.a.	n.a.	45.2 ± 8.0	39.1 ± 7.6 (*n*=62)
125.3	n.a.	11.40%	42.11	n.a.
109.80 ± 17.9	n.a.	15.05 ± 6.0%	37.27 ± 4.9	37.47 ± 5 (*n*=39)
124.7 ± 22.6	28.40%	11.80%	42.8 ± 7.0	37.88 ± 6.7
115.6 ± 21.1	n.a.	12.99%	40.0 ± 5.8	n.a.
125.0 ± 21.7	33.8 ± 20.9%	15.90%	43.4 ± 6.5	37.9 ± 6,8 (*n*=65)
115 ± 21	n.a.	11.7 ± 7.1%	39 ± 6	34.8 ± 3.2
109.93 ± 17	43.6 ± 16%	17.20%	42.19 ± 5	43.6 ± 16 (*n*=72)
n.a.	10.20%	n.a.	48.8 ± 8.5kg	n.a.
n.a.	46 ± 18%	n.a.	43 ± 5.6	n.a.
119.2 ± 22.9	39.0 ± 3.9%	n.a.	44.8 ± 7.4	38.1 ± 0.7 (*n*=13)
118.1 (109.8–125, *n*=632)	33.5% (10.2–46%, n=500)	13.72% (11.4–17.2%, *n*=610)	42.6 (37.3–48.8)	38.4 (34.8–43.6, *n*=600)
Weight loss outcomes of PPBS				
Mean weight in kg at baseline	EWL% at 1 year	TWL% at 1 year	BMI in kg/m2 at baseline	BMI in kg/m2 at 1 year
123.4 ± 20	69.2 ± 16.4% (*n*=62 )	34.6 ± 9.2% (*n*=62)	43.2 ± 5.7	27.9 ± 3.2
142.64	n.a.	n.a.	49.94	n.a.
142.65 ± 30.83	77.69 ± 20.92% (*n*=266 )	n.a.	49.8 ± 8.8	31.8 ± 5.48
n.a.	62.4% (*n*=58)	37.1 ± 6.6% (*n*=58)	57.3 ± 9.2	35.3 ± 5.8
119.5	91%	39%	44.3	n.a.
n.a.	81.94 ± 9.51%	n.a.	48.28± 3.80	28.19 ± 2.14
132 (119.5– 142.65, *n*=1294)	76.45 (62.4–91%, *n*=582)	36.9% (34.6–39%, *n*=288)	48.8 (43.2–57.3)	30.8(27.9–35.3, *n*=696)

Number of patients is presented separately where it differed from the overall number. Range and SD were added where they were available and where necessary

**Table 5:** Comparison of patient characteristics between groups

DJBL	Number of publications	Overall number of patients	Death	Early removal	Device-related AE rate	Severe events	Mean weight in kg at baseline	EWL% at 1 year	TWL% at 1 year	BMI in kg/m2 at baseline	BMI in kg/m2 at 1 year
	11	800	0	152 (19%)	588 (73.5%)	155 (19.4%)	118.1 (109.8–125, *n*=632)	33.5% (10.2–46%, *n*=500)	13.7% (11.4–17.2%, *n*=610)	42.6 (37.3–48.8)	38.4 (34.8–43.6, *n*=600)
PPBS	Number of publications	Overall number of patients	Death	Reoperation	Overall number of complications	Severe complications	Mean weight in kg at baseline	EWL% at 1 year	TWL% at 1 year	BMI in kg/m2 at baseline	BMI in kg/m2 at 1 year
	6	1462	4 (0.3%)	37 (2.5%)	182 (12.4%)	84 (5.7%)	132 (119.5–142.65, *n*=1294)	76.5% (62.4–91%, *n*=582)	36.9% (34.6–39%, *n*=288)	48.8 (43.2–57.3)	30.8(27.9–35.3, *n*=696)

Number of patients is presented separately where it differed from the overall number. Range is added where available and necessary.

### Synthesis of results

**Fig. 2:**
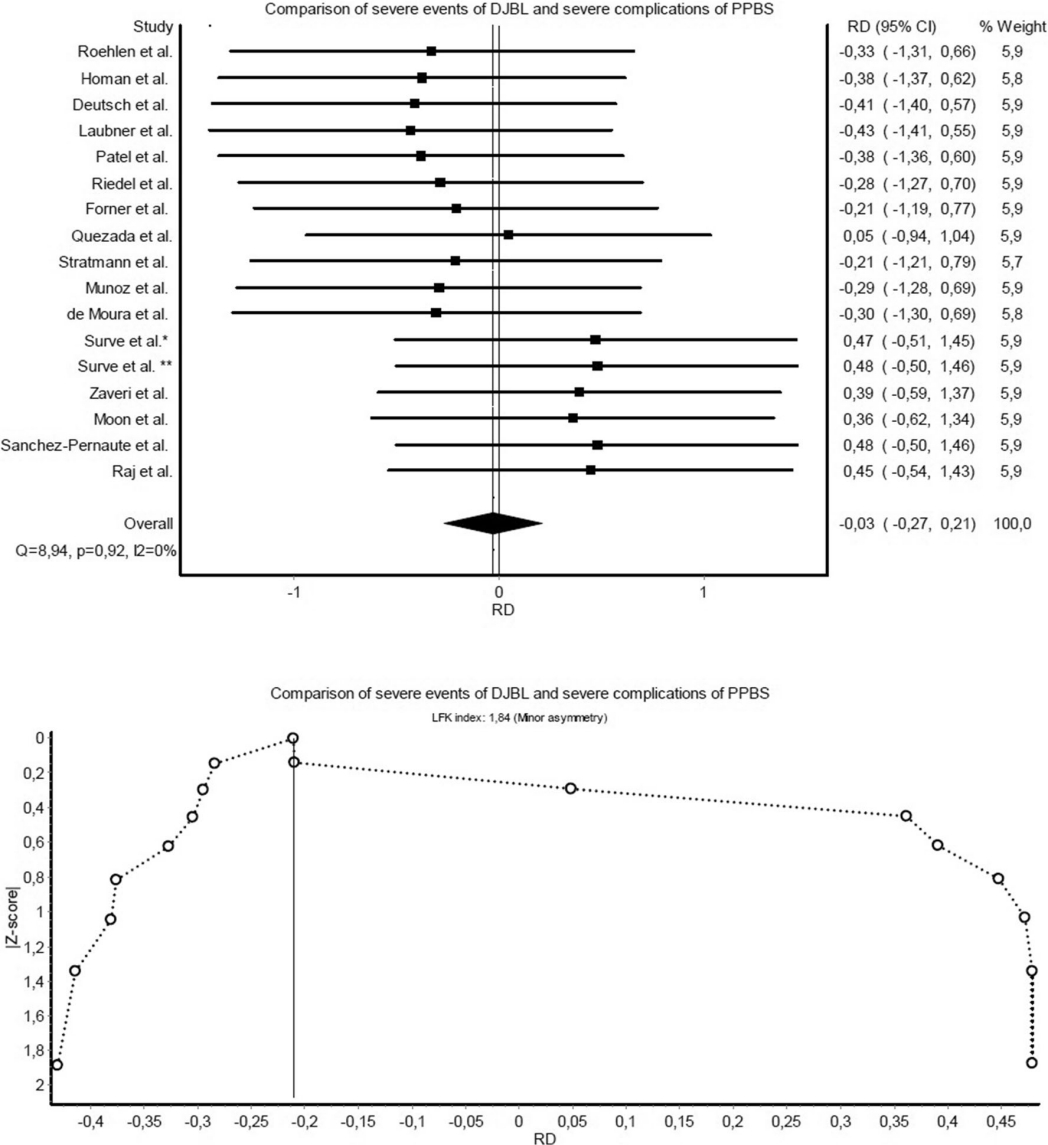
Forest and Doi plot of meta-analysis comparing author-defined severe events of DJBL and author-defined severe complications of PPBS

**Fig. 3:**
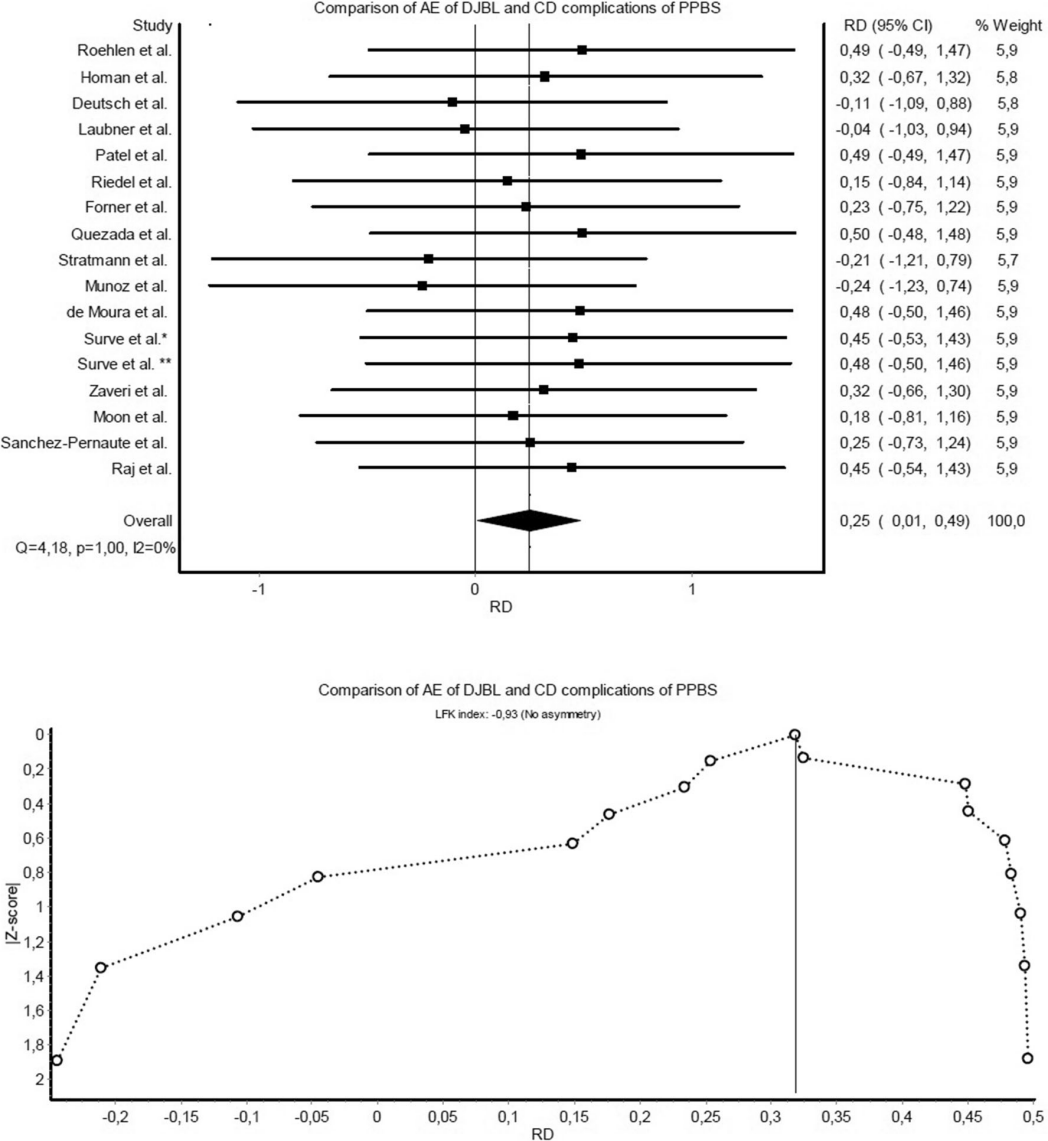
Forest and Doi plot of meta-analysis comparing AEs of DJBL and CD complications of PPBS

**Fig. 4:**
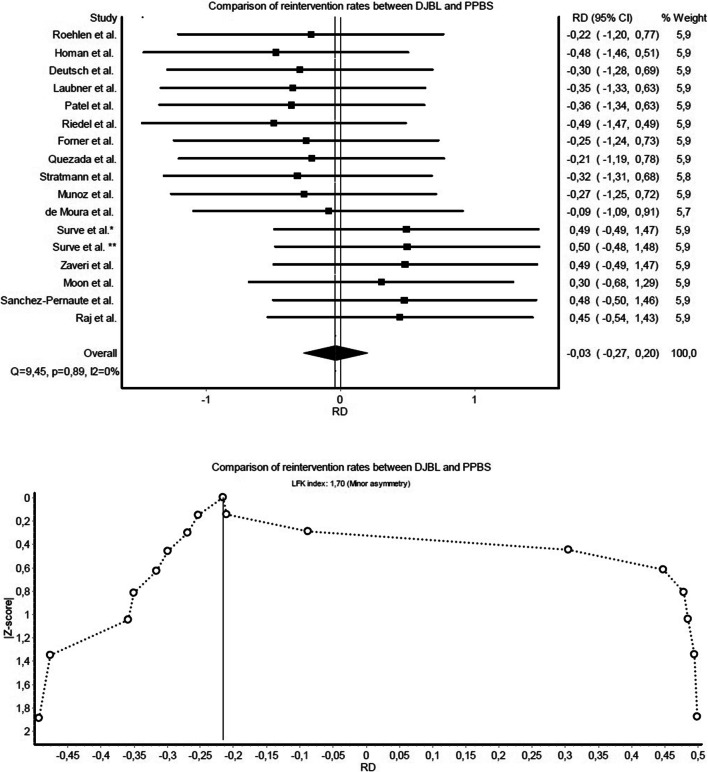
Forest and Doi plot of meta-analysis comparing reintervention rates

**Fig. 5:**
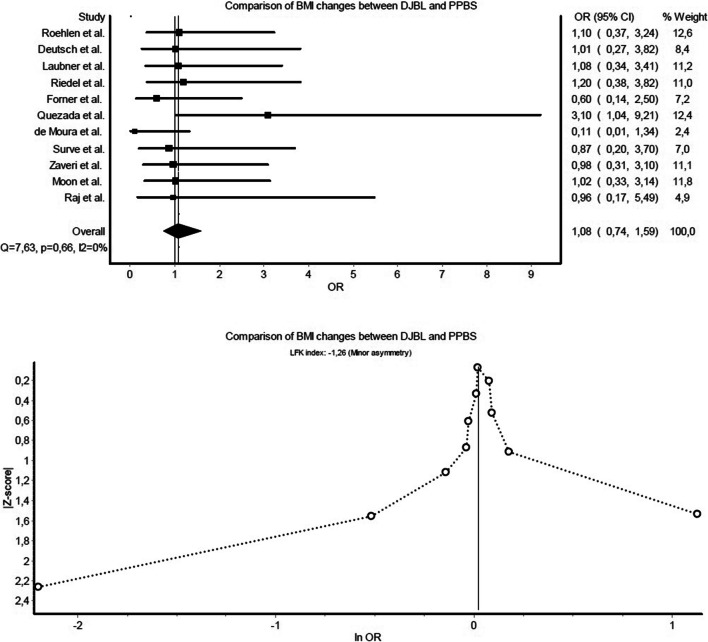
Forest and Doi plot of meta-analysis comparing changes in BMI. Sample size consisted of 599 and 414 cases for DJBL and PPBS, respectively.

Comparison of DJBL-related severe events and PPBS-related severe complications defined by authors (Fig. 2) showed almost equal risk (RD: −0.03, CI: −0.27 to 0.21). Regarding overall AE and CD complications (Fig. 3), PPBS was superior to DJBL due to an excess risk of 25% (RD: 0.25, CI: 0.01–0.49). Reintervention rates (Fig. 4) were similar (RD: −0.03, CI: −0.27 to 0.20). For weight loss outcomes, changes of BMI (Fig. 5) were compared and indicated similar efficacy for both investigated methods (OR: 1.08, CI: 0.74–1.59).

### Risk of bias across studies

In comparisons of DJBL-related severe events and PPBS-related severe complications, studies were homogenous (*Q*=8.94, *p*=0.92, *I*^2^=0%), and Doi plot (Fig. 2) warranted only a minor risk for publication bias (LFK index: 1.84). Regarding AEs and CD complications, there was no heterogeneity proven (*Q*=4.18, *p*=1.00, *I*^2^=0%), and the Doi plot (Fig. 3) showed no asymmetry (LFK index: −0.93). Studies were also homogenous when comparing reintervention rates (*Q*=9.45, *p*=0.89, *I*^2^=0%), with a minor risk of publication bias (LFK index: 1.70) (Fig. 4). There was no heterogeneity observed in the meta-analysis of BMI changes (Fig. 5), and the risk of publication bias was minor for weight loss outcomes (LFK index: −1.26).

## Discussion

### In general

The aim of restrictive procedures is to decrease stomach volume. If the fundus is removed, satiety will emerge faster and will last longer because of lowered ghrelin levels [31]. Duodeno-jejunal exclusion results in more complex effects of gut hormones. Changes in cholecystokinin (CCK) and protein Y mechanism affect satiety. Incretins (mainly glucagon like peptide 1 (GLP1)) influence serum glucose levels by antagonizing glucagon [32–34], and the latter effects make duodeno-jejunal exclusion more efficient in weight loss management and metabolic improvement compared to solely restrictive procedures. The mechanism is independent of the type of procedure applied (gastric bypass methods or PPBS).

### Summary of evidence

Safety is the most important thing when introducing a novel method. DJBL has been regarded as being safer than bariatric surgery [35, 36]. Our opinion is that it is essential to preserve the function of the pylorus; therefore, we decided to compare this method to PPBS as a control group, because DJBL is theoretically regarded as mimicking duodeno-jejunal exclusion. SADI-SG is more frequently represented in the literature than SADJ-SG. The length of the afferent limb should affect complications and efficacy, but such a statement has not yet been proven well. Surprisingly, our meta-analysis found a higher risk of DJBL-related AEs compared to PPBS-related CD complications. Authors found fewer than expected mild to moderately severe (CD1-2) complications (such as GIT events, malabsorption, diarrhoea) reported for PPBS. The reason could be due to inaccurate publishing of such complications by some of the studies in the surgery group. It should be taken into consideration that cumulative mortality was zero after DJBL implantation, while four patients died in the surgical group (only one case was directly related to the intervention). Reintervention rates were unexpectedly similar between the two methods. After DJBL, early device removal was the most frequent type of reintervention. After any kind of bariatric surgery intervention, there could be various reoperations due to different indications, and there was no difference between the groups in this aspect. Each method was efficient regarding weight loss outcomes, without significant differences, yet more favourable weight management could be achieved by applying PPBS. The DJBL is usually in place for 6 months (which could be extended to 12 months), resulting in an increase in body weight after explantation, while metabolic parameters worsen. As for future prospects, we hope that the implantation period could be extended to achieve an even better outcome. After publishing long-term data on temporary metabolic procedures, we will be able to compare them with purely surgical methods in order to obtain more precise guidelines. We must emphasize the disadvantage caused by the increasing difficulty of reverting any kind of bariatric surgery (especially when part of the upper GIT is bypassed) to normal anatomy, compared to endoscopic interventions.

### Comparison with other procedures

LRYGB dominated bariatric surgery for a long period of time. Later, it was replaced by LSG due to its greater simplicity and more favourable efficacy. Perioperative mortality rates are incredibly low (below 0.2%), and the rates of overall serious complications are lower than 6% for LSG and 9% for LRYGB, respectively. Short-term reoperation rates should be kept below 3% for LSG and 5% for LRYGB. The long-term TWL% of each method is around 20% [37]. The latest systematic reviews including meta-analysis showed controversial results in terms of efficacy and safety when comparing LYRGB with LSG [38–40]. OAGB was proven to be effective and safe compared to LYRGB [41, 42]. Our results are comparable to former studies on widespread bariatric surgery procedures. PPBS-related mortality (0.3%), reoperations (2.5%) and severe complications (5.7%) are comparable to widely used metabolic interventions. In contrast, there was zero mortality in the DJBL group, yet 19% of implanted devices were removed earlier, and more severe AEs (19.4%) were observed. PPBS represents similar weight loss outcomes to LYRGB, SAGB or LSG; however, DJBL provides less favourable results.

### Limitations

Our review has limitations, as the included studies lack RCTs. There is a minor to moderate risk of publication bias. DJBL is a temporary method, contrary to PPBS, which has long-term efficacy; therefore, comparison could be ambiguous. While DJBL is regarded as mimicking DS procedures, it seems to be more practical to compare it to the gold standard pylorus preserving duodeno-jejunal bypass, despite emerging concerns. Due to the lack of long-term data on the efficacy of DJBL, short-term (1-year implantation period) results were compared to the surgical group to achieve more relevant results. SADI-SG seems to provide a more hypoabsorptive effect than SADJ-SG, but there are no strong recommendations supporting this. Definitions of severe AEs varied between DJBL trials, which could confuse our results. Thus, we decided to determine which AEs were classified as severe to achieve a more accurate comparison. Mild to moderate surgical complications, especially the most frequent late side effects (malabsorption and diarrhoea), were underrepresented in the papers involved, which could skew our results. In addition, the published parameters of weight loss were not unified, which reduced the value of the comparison.

### Conclusion

Only limited conclusions can be made based on our findings. PPBS was superior to DJBL with regard to safety outcomes (GRADE IIB), which failed to support the authors’ hypothesis. Surgical procedures showed lower complication rates than the incidence of DJBL-related AEs, although it should be emphasized that the low number of PPBS-related mild to moderate complications reported could be the result of incomplete data recording in the analysed publications. Weight loss outcomes were in favour of bariatric surgery (GRADE IIB). As the DJBL is implanted into the upper GIT for 6 to 12 months, it seems a promising additional method in the inventory of metabolic interventions.

## Abbreviations

AE: Adverse event
BMI: Body mass index
CCK: Cholecystokinin
CD: Clavien-Dindo classification
CI: Confidence interval
DJBL: Duodeno-jejunal bypass liner
DS: Duodenal switch
EWL%: Percent of excess of weight loss
FDA: Food and Drug Administration
GIT: Gastrointestinal tract
HgbA1C%: Haemoglobin A1C in percent
LSG: Laparoscopic sleeve gastrectomy
LRYGB: Laparoscopic Roux-en-Y gastric bypass
OAGB: One-anastomosis gastric bypass
PPBS: Pylorus preserving bariatric surgery
RCT: Randomized controlled trial
SADI-SG: Single-anastomosis duodeno-ileal bypass with sleeve gastrectomy
SADJ-SG: Single-anastomosis duodeno-jejunal bypass with sleeve gastrectomy
SD: Standard deviation
T2DM: Type 2 diabetes mellitus
TWL%: Percent of total weight loss
RD: Risk difference
OR: Odds ratio
VTE: Venous thromboembolism
